# Retraction Note to: Slug down-regulation by RNA interference inhibits invasion growth in human esophageal squamous cell carcinoma

**DOI:** 10.1186/s12876-021-01971-7

**Published:** 2021-10-22

**Authors:** Peng Tang, Zhentao Yu, Kejun Zhang, Yu Wang, Zhongliang Ma, Shaoyan Zhang, Dong Chen, Yanbing Zhou

**Affiliations:** 1 Department of Esophegeal Oncology, Key Laboratory of Cancer Prevention and Therapy, Cancer Institute and Hospital of Tianjin Medical University, Tianjin, 300060 People’s Republic of China; 2 Department of Surgery, The Affiliated Hospital of Medical College, QingDao University, QingDao, 266003 Shan Dong Province People’s Republic of China; 3 Department of Laboratory, the Affiliated Hospital of Medical College, QingDao University, QingDao, 266003 Shan Dong Province People’s Republic of China

## Retraction Note: BMC Gastroenterol (2011) 11:60 10.1186/1471-230X-11-60

The Editor has retracted this article after concerns were raised regarding the figures and the contents of the article, including:

- Inaccurate description of results in the main text and figure legends;
- Inconsistencies between the descriptions of results and figures;
- Overlap between images in Figs. 4a, b (western blot) and 6a, b (cell images);
- Issues with western blot images presented in Figs. 3 and 4.

The Editor no longer has confidence in the results and conclusions presented.

None of the authors have responded to correspondence from the editor or publisher about this retraction.

